# A supplemental screw enhances the biomechanical stability in medial open-wedge high tibial osteotomy

**DOI:** 10.1371/journal.pone.0244557

**Published:** 2020-12-30

**Authors:** Jesse Chieh-Szu Yang, Philipp Lobenhoffer, Chia-Ming Chang, Cheng-Fong Chen, Hsiu-Chen Lin, Hsuan-Hsiao Ma, Pei-Yuan Lee, Oscar Kuang-Sheng Lee

**Affiliations:** 1 Department of Orthopedics and Traumatology, Taipei Veterans General Hospital, Taipei, Taiwan; 2 Institute of Clinical Medicine, National Yang-Ming University, Taipei, Taiwan; 3 Sportsclinic Germany, Hannover, Germany; 4 Department of Physical Therapy and Graduate Institute of Rehabilitation Science, China Medical University, Taichung, Taiwan; 5 Department of Physical Therapy and Assistive Technology, National Yang Ming University, Taipei, Taiwan; 6 Department of Orthopedics, School of Medicine, National Yang-Ming University, Taipei, Taiwan; 7 Orthopedic Department, Show Chwan Memorial Hospital, Changhua, Taiwan; 8 College of Nursing and Health Sciences, DAYEH University, Changhua, Taiwan; 9 Department of Orthopedics, China Medical University Hospital, Taichung, Taiwan; Assiut University Faculty of Medicine, EGYPT

## Abstract

**Background:**

The supplemental screw technique was introduced for salvage of lateral hinge fracture in medial open-wedge high tibial osteotomy (owHTO). The efficacy of its use in protection of lateral hinge fracture and corresponding biomechanical behaviors remained unclear. The current study was aimed to clarify if a supplemental screw can provide better protection to lateral hinge in biomechanical perspective.

**Materials:**

An in vitro biomechanical test was conducted. Tibial sawbones, commercial owHTO plates and a cannulated screw were utilized for preparing the intact, owHTO, and owHTO with cannulated screw insertion specimens. A “staircase” dynamic load protocol was adopted for axial compressive test with increasing load levels to determine structural strength and durability by using a material testing system, while a motion capture system was applied for determining the dynamic changes in varus angle and posterior slope of the tibia plateau with various specimen preparation conditions.

**Results:**

Type II lateral hinge fracture were the major failure pattern in all specimens prepared with owHTO. The insertion of a supplemental cannulated screw in medial owHTO specimens reinforced structural stability and durability in dynamic cyclic loading tests: the compressive stiffness increased to 58.9–62.2% of an intact specimen, whereas the owHTO specimens provided only 23.7–29.2% of stiffness of an intact specimen. In view of tibial plateau alignment, the insertion of a supplemental screw improved the structural deficiency caused by owHTO, and reduced the posterior slope increase and excessive varus deformity by 81.8% and 83.2%, respectively.

**Conclusion:**

The current study revealed that supplemental screw insertion is a simple and effective technique to improve the structural stability and durability in medial owHTO.

## Introduction

The concept of lower limb realignment by medial open-wedge high tibial osteotomy (owHTO) has been widely accepted for the treatment of medial compartment osteoarthritis and varus deformity of knee joint [1–3], especially for young patients or active seniors to postpone the necessity of total knee arthroplasty [4–7]. By medial opening of the proximal tibia, correction of mechanical alignment and load redistribution on the tibial plateau can be easily achieved without approaching the fibula or the superior tibiofibular joint [8]. In order to enhance rotational stability after medial owHTO surgery, a classical biplanar osteotomy technique which preserves bony anterior buttress was introduced in 2003 [9].

The risk of alteration of the posterior slope of the tibial plateau has been reported in several clinical studies [10–13]. Keeping the lateral hinge intact is very important for bone healing after medial owHTO surgery [14]. However, due to structural deficiency after surgery, loss of reduction, bone screw breakage, and lateral hinge fracture are common complications that cause failure of surgery [15–17]. According to Takeuchi’s classification of proximal tibial hinge fractures associated with medial owHTO, the type II fracture where the fracture line extends downwards from the lateral hinge may jeopardize the structural stability, causes severe pain and over correction of coronal alignment of the knee joint [18]. Once a lateral hinge fractures occurs, the technique of using supplemental screw fixation from the lateral cortex to the medial tibial plateau may be helpful to restore angular correction [19] with the support of biomechanical validation [20]. In view of structural stability, the finite element study by Yang et al. has further confirmed the contribution of supplemental cannulated screw insertion in reducing the reduction loss of the posteromedial tibial plateau, without increasing the risk of implant damage [21]. Clinical feasibility for the use of the “extra focal” supplemental screw has also been demonstrated by Khalifa et al. in the medial owHTO surgery [22].

Previous studies have confirmed that supplemental screw insertion plays a successful role in salvage of lateral hinge fracture and partially restores biomechanical stability in medial owHTO cases [19, 20]. However, the efficacy of supplemental screw insertion for lateral hinge protection was not clarified yet. Therefore, the purpose of the current study was to further verify if the supplemental screw insertion technique can be applied as a protective strategy to lateral hinge fracture. Practical biomechanical tests were conducted for gathering quantified information.

## Materials and methods

### Specimen preparation

**Fig 1:**
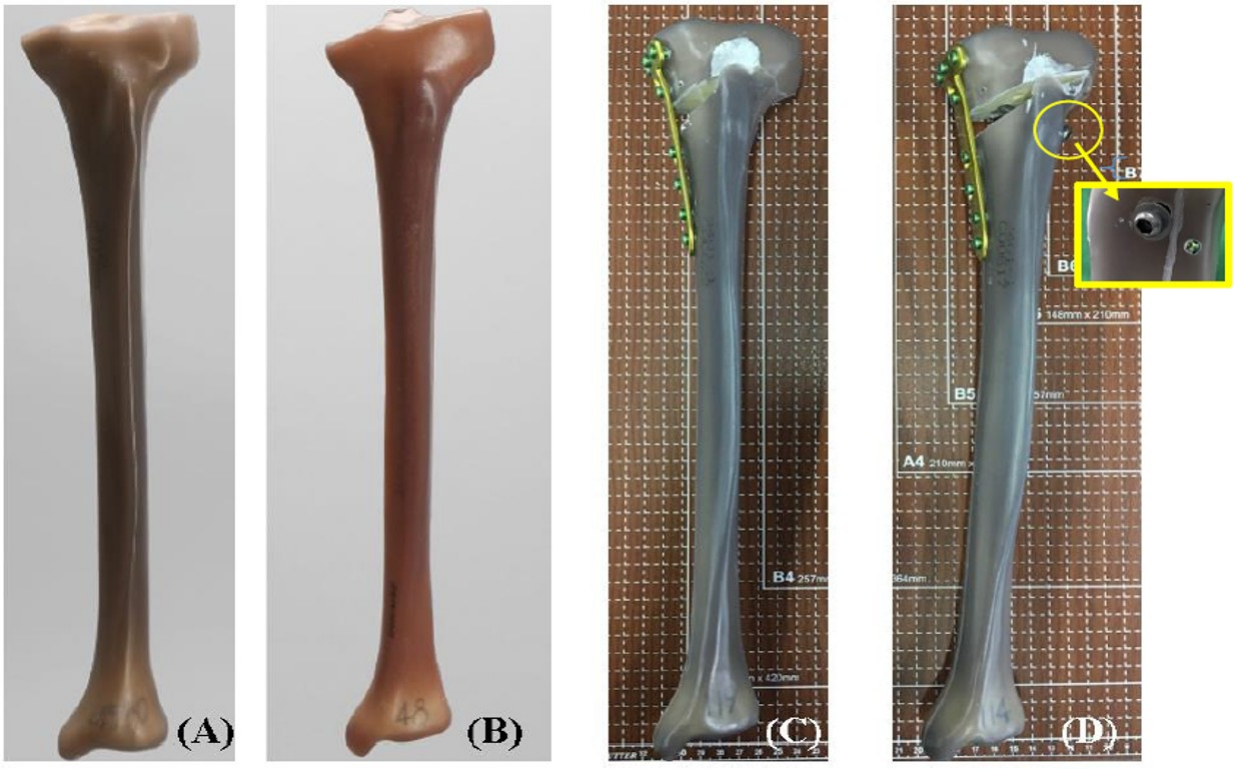


For purpose of standardization, 4^th^ generation sawbones were utilized in the current study [23, 24]. A total of 7 left tibial sawbones including 1 normal (Model 3401, tibia, 4^th^ generation, composite, medium; A Pacific Research Company, WA, USA) and 6 standard varus (Model 3401–1, tibia, 4^th^ generation, composite, with varus deformity, medium) models were used. The 6 varus models were treated by a single experienced surgeon, with medial owHTO referring to the standard TomoFix Osteotomy System Surgical Guide, and corrected by 10 degrees to valgus using a bi-planar osteotomy technique. TomoFix Medial High Tibial Plates (DePuy Synthes, Synthes GmbH, Oberdorf, Switzerland) and locking screws were implanted for fixation. Three specimens were randomly selected from the 6 varus-correction models. An 8-mm cannulated screw (Stryker Corporation, Kalamazoo, MI, USA) and washer was additionally placed in these specimens (supplemental screw). Referring to a previous study by Yang et al., the supplemental cannulated screw was inserted from the lateral cortex to the region beneath the medial-lateral tibial plateau, in an orientation of approximately 50° oblique in the coronal plane and 38.5° oblique in the transverse plane [21]. A specialized targeting jig was used for drilling the screw hole to reduce the supplemental screw insertion differences among the 3 specimens. The specimens were then categorized into 3 groups: intact (n = 1), models corrected with the TomoFix bone plate only (group 1, n = 3), and models corrected with the TomoFix bone plate plus reinforcement with the supplemental screw (group 2, n = 3) (Fig 1).

All specimens were embedded into a bone cement mould at the distal region. Before the embedding procedure, the distal tibia was shortened by 10 cm to remove the medial malleolus region. Using the cannulated shaft axis of the tibial sawbone as reference, the specimen was place into an aluminium cylindrical mould with a central vertical pole. The cannulated tibial shaft was placed on the pole and the mould filled with commercial bone cement to have an 8-cm embedment. All embedded specimens were placed in room temperature for 48 hours for fully cement polymerization.

### Test settings

**Fig 2:**
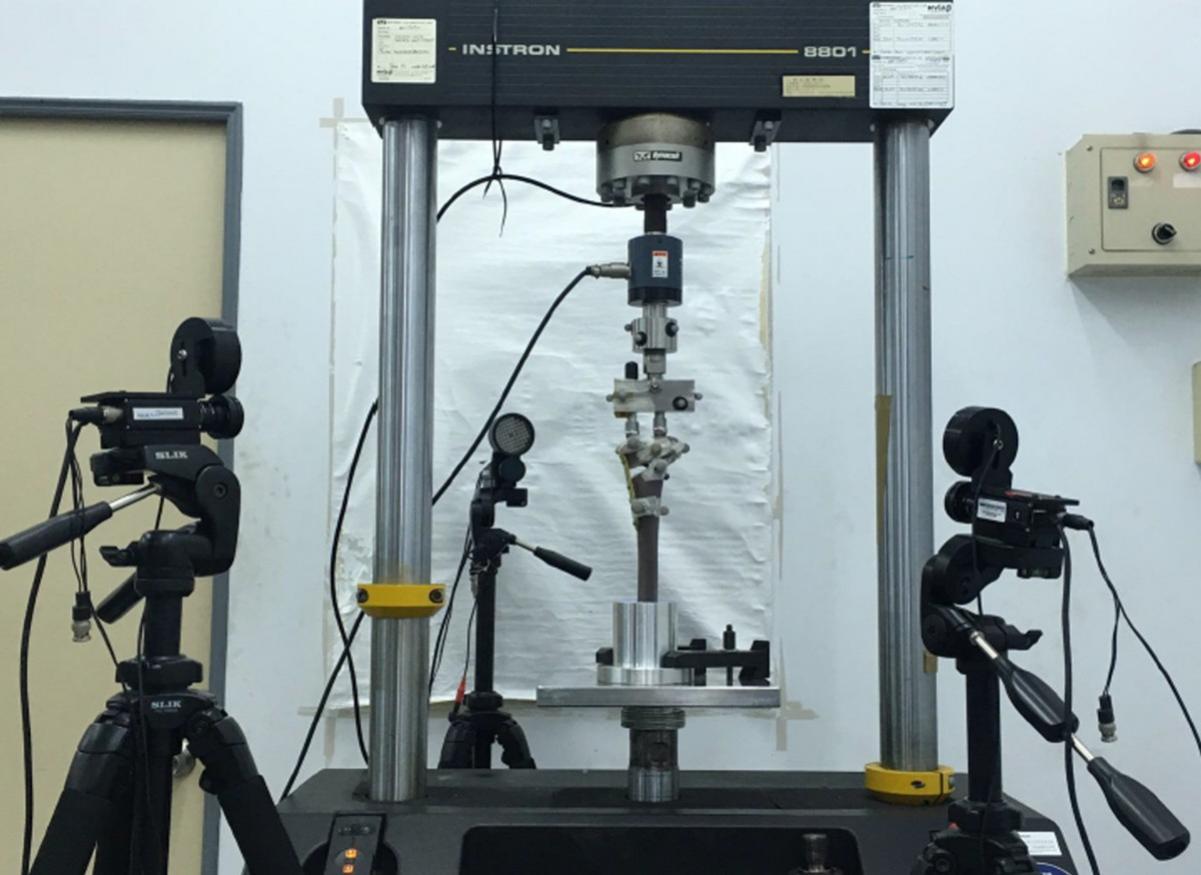


**Fig 3:**
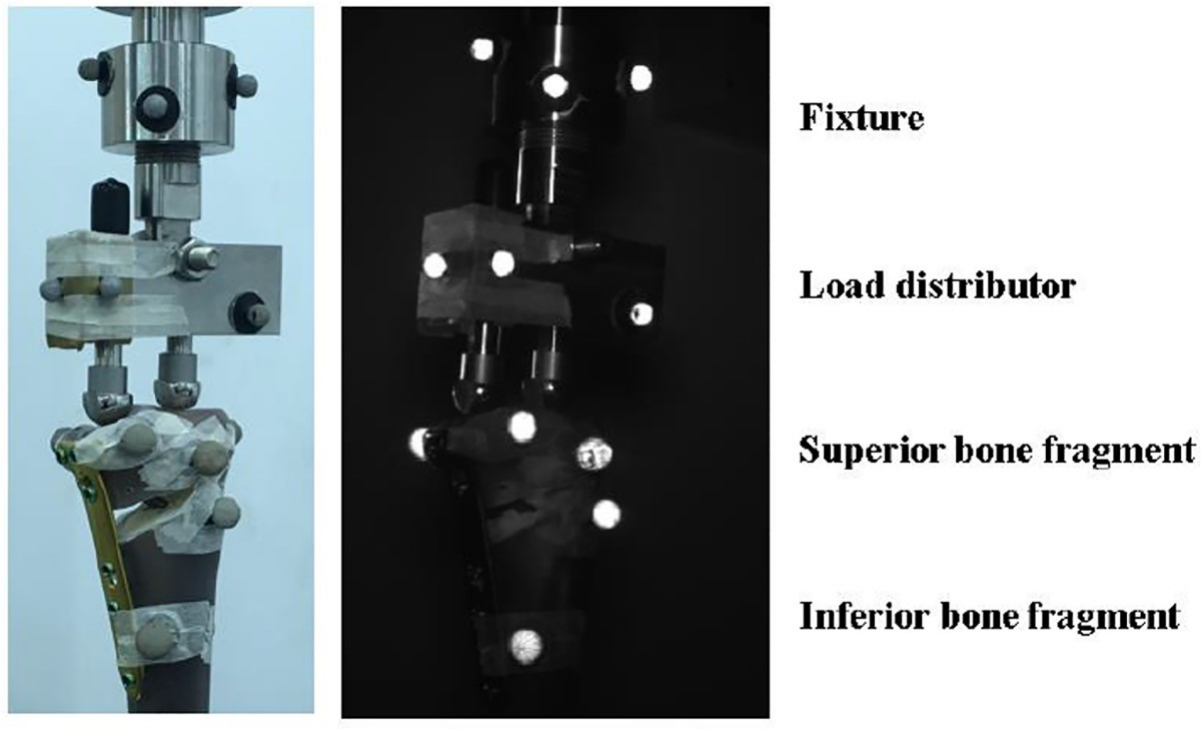


An Instron 8801 Material Testing System (Instron, Norwood, MA, USA) was used for the mechanical tests. The test specimen was fixed at the base of testing machine, with the tibial shaft axis vertical to the ground. Compressive load was exerted onto both medial and lateral condyles of tibial plateau, through a hinged load distributor to permit a balanced resultant force to be located on the tibial plateau with a 62% lateral offset as described in previous in vitro studies [24, 25], simulating the Fujisawa point [26]. In order to acquire detailed information of tibial plateau movement, the motion system MaxTRAQ (Innovision System Inc., Marietta, GA, USA) was utilized for capturing the motion at two major (sagittal and coronal) planes to determine the stability at the osteotomy site. In the current study, the optical system consisted of three CCD-cameras (Fig 2) and multiple optical markers (Fig 3) to define the basis (fixture, load distributor, superior bone fragment, and inferior bone fragment) for further mathematical calculation.

### Mechanical test protocol

According to in vitro test protocol for medial owHTO by Agneskirchner et al. [25], a preload of 50 N was first exerted on the specimen as the initial load, followed by a 0.25 Hz sinusoidal cyclic load of 150 to 800 N for 60 cycles. After stabilization of the test specimen, the incremental cyclic protocol was completed until test termination. The initial magnitude of compressive load was 50–800 N at 2Hz. The maximal load was then increased by 160 N after completing every 10,000 cycles, while the minimal load was kept at 50 N (Table 1) [24, 25]. For every 1,000 cycles, the CCD-cameras captured the motion of specimens for 10 seconds with a sampling rate of 20 frames per second. Terminal criteria of the test included the observation of cortex fracture, an axial compressive displacement exceeding 2.5 mm. A total of 9 load levels (90,000 load cycles) were accomplished if no specimen failure occurred.

**Table 1:** Description of load level in the current study.

Load level	Magnitude	Cycle counts
1^st^	50–800 N	0–9,999
2^nd^	50–960 N	10,000–19,999
3^rd^	50–1,120 N	20,000–29,999
4^th^	50–1,280 N	30,000–39,999
5^th^	50–1,440 N	40,000–49,999
6^th^	50–1,600 N	50,000–59,999
7^th^	50–1,760 N	60,000–69,999
8^th^	50–1,920 N	70,000–79,999
9^th^	50–2,080 N	80,000–89,999

#### Data acquisitions and evaluation

The reference coordinate system was defined by the tibial shaft axis (Z axis, in accordance with the direction of compression load, perpendicular to the ground), anteroposterior axis (Y axis, parallel to the ground), and the mediolateral axis (X axis). The load-displacement data for all specimens were recorded by the built-in program of the Material Testing System, representing the structural performance at the Z axis. The compressive stiffness of the specimen was calculated by the cyclic load over the axial displacement (between peak and valley locations of each cycle), averaged for each load level. After labeling the optical makers in the program provided by MaxTRAQ, the tibial plateau slope changes were defined by the relative angular movement between the superior and inferior fragments in coronal plane (the X-Z plane) and sagittal plane (the Y-Z plane), calculated from the videos recorded by the CCD-cameras, averaged for each load level as well. Positive values of the slope changes represent increased varus (in coronal plane) or increased posterior slope (in sagittal plane) as signs of reduction loss. Three testing groups (intact, group 1and 2) were compared.

## Results

### Durability of test specimens

Due to difficulty in determining the exact failure cycle during the test, the durability of the test specimen was represented in a 100-cycle interval (Table 2). Among all tested specimens, only the intact group has completed the full 9 load levels, passed 90,000 load cycles without any observable cortex damage or excessive axial displacement. All specimens in the group 1 and 2 did not accomplish the full test, but represent type II hinge fracture at the lateral cortex beneath the hinge, determined as the termination of the test. No specimen reached the terminal criteria where a 2.5 mm axial displacement was detected. No implant failure was observed in all instrumented specimens.

**Table 2:** Durability comparison for the three test groups.

Test group	Specimen #	Last load level	Durability
intact	1	9	Passed 90,000 cycles (no failure)
Group 1	1	4	37,700–37,800 cycles
	2	4	36,800–36,900 cycles
	3	4	38,000–38,100 cycles
Group 2	1	6	52,900–53,000 cycles
	2	6	52,900–53,000 cycles
	3	7	62,300–62,400 cycles

### Compressive stiffness

**Fig 4:**
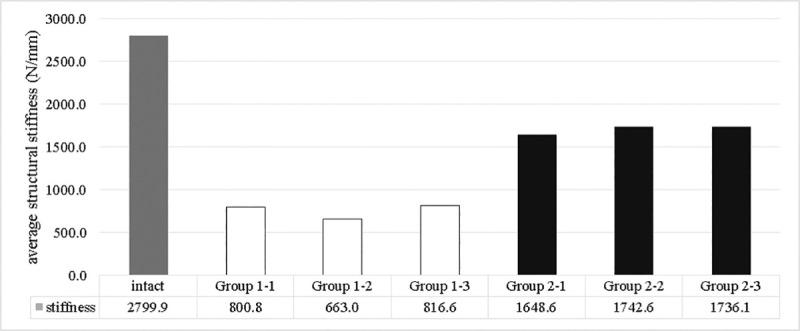


Quantified structural compressive stiffness is represented in Fig 4. The group 1 preserved only 23.7–29.2% of the strength of the intact group. By adding the supplemental screw, the group 2 improved the strength to 58.9–62.2% of the intact group.

### Changes in tibial plateau slopes

**Fig 5:**
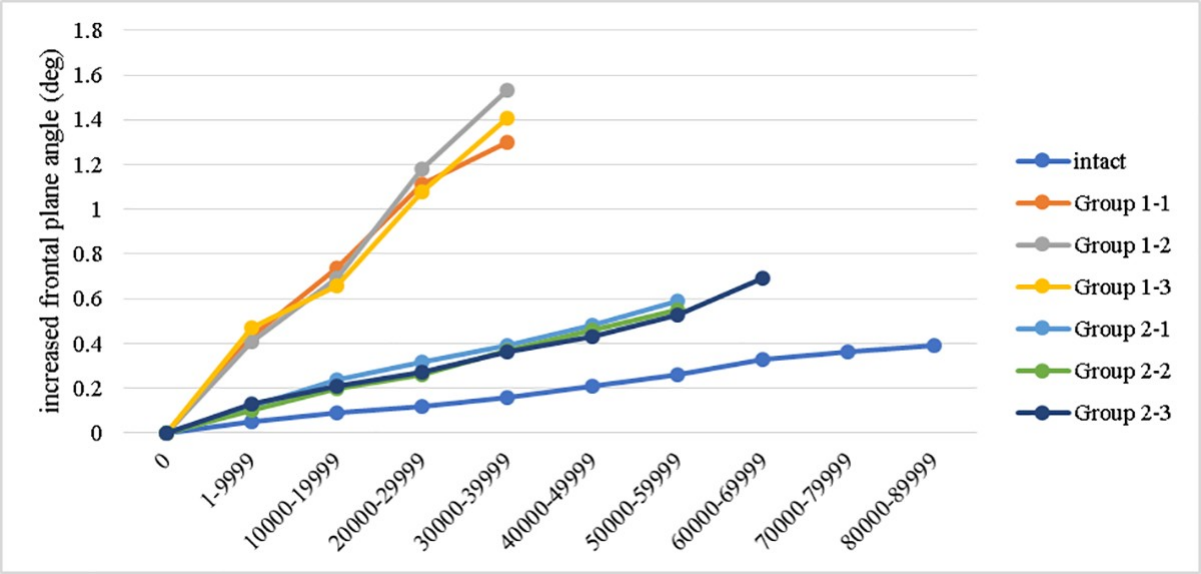


Fig 5 shows the changes of the tibial plateau slope in coronal plane (varus angle). The intact group represented the lowest slope change to 0.39 degrees at the end interval of load levels. The group 1 showed the greatest varus deformity among the three groups. At the 4^th^ load level (endpoint of the group 1), the increased varus angle for intact, group 1, and group 2 were respectively 0.16, 1.41±0.12 (range 1.3–1.53), and 0.37±0.01 (range 0.36–0.39) degrees. At the 6^th^ load level (endpoint of specimens 1 and 2 for group 2), the increase of varus angle were 0.26 and 0.56±0.03 (range 0.53–0.59) degrees. The 3^rd^ specimen of the group 2 showed an increased varus angle to 0.69 at the endpoint of the 7^th^ load level.

**Fig 6:**
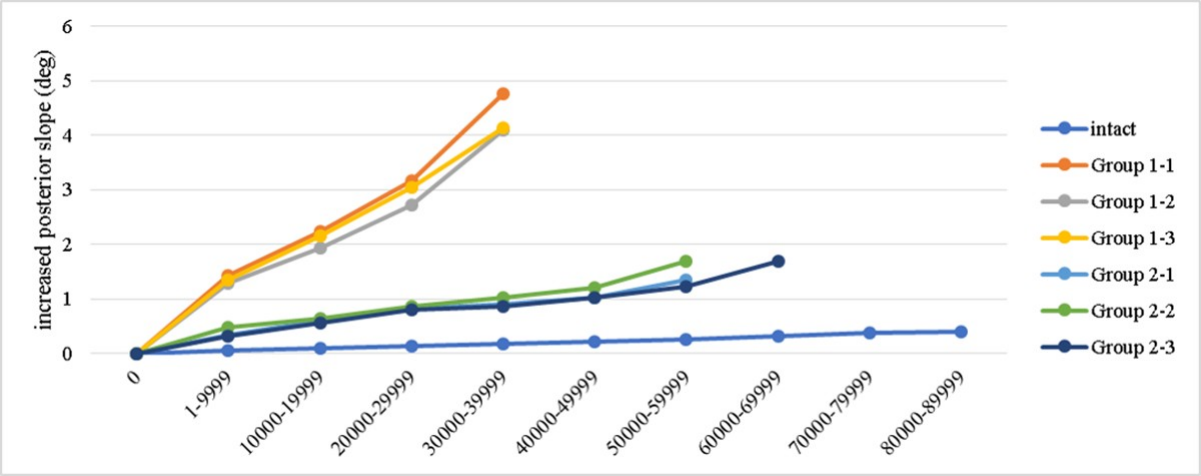


Fig 6 shows the change of tibial plateau slope in sagittal plane. At the 4^th^ load level the increase of posterior slope for intact, group 1, and group 2 groups were respectively 0.17, 4.34±0.38 (range 4.1–4.77), and 0.93±0.09 (range 0.85–0.91) degrees. At the 6^th^ load level, the increase of posterior slope for intact group and group 2 were 0.263 and 1.42±0.23 (range 1.23–1.68) degrees. The 3^rd^ specimen of group 2 showed an increased posterior slope to 1.68 degrees at the 7^th^ load level (endpoint), while the slope of the intact specimen was 0.32 degrees.

## Discussion

As a strategy for postponing the necessity of total knee arthroplasty for patients with varus deformity or medial compartment osteoarthritis, the medial owHTO must take the issue of joint stability into serious concern. In the concept of enhanced recovery after surgery (ERAS), it will be very beneficial to bone healing that patients can return to the original activity level and weight bearing. Early full-weight bearing with adequate interfragmental stability may improve the early clinical outcome for patients who receive owHTO surgery [27]. If the implant cannot offer sufficient stability, the delayed or nonunion of osteotomy site [28, 29] causing structural instability may lead to the aforementioned complications [15–17]. Usage of supplemental screw as a salvage of lateral hinge fracture has been reported [19, 20], with evidence of clinical feasibility in medial owHTO surgery [22]. The current study has validated that a supplemental screw insertion can reinforce the stability and durability of a medial owHTO structure, compared to the situation with a bone plate for proximal medial tibia fixation alone. The quantified biomechanical data may enhance the confidence and motivation for surgeons who will conduct knee preservation surgeries.

Biomechanical methods are commonly applied in studying the in vitro stability and durability of osteotomy models. For simplifying the test for determining structural stability and durability under different loading magnitudes, Agneskirchner et al. have firstly introduced a “staircase” dynamic loading protocol to represent the biomechanical behavior of owHTO structures with various designs of owHTO implants. A linear displacement transducer was applied to measure the gap motion at the osteotomy site [25]. In their study, a long rigid bone plate was superior to short plate-wedge designs for stability. Following this study, Schröter et al. conducted another biomechanical study by applying the staircase dynamic loading on TomoFix bone plates on medial owHTO models, and compared the usage of conventional rigid locking screws and dynamic locking screws [24]. The optical motion system was applied in their study to acquire the movement of bone fragments. Results revealed that no owHTO specimen could pass the full 9 load levels without the occurrence of lateral hinge fracture. The rigid locking screws represented better durability in cyclic loading and greater structural stability, while dynamic locking screws were more flexible to provide the required interfragment movement that may be beneficial to bone healing. For the supplemental screw fixation technique in medial owHTO, Freitas et al. have conducted a series of biomechanical test and have clarified that the extra supplemental screw fixation indeed enhanced the structural stability for lateral hinge fractured models [20]. As a protective strategy to lateral hinge fracture, the group 2 in the current study showed better durability in the staircase dynamic loading test, sustained to 6^th^ to 7^th^ load level until the occurrence of type II hinge fracture. The group 1 showed comparatively lower durability to 5^th^ load level. A stress-based biomechanical study has discussed about the strategy in avoidance of hinge fracture during gap expansion by pre-drilling a hole at the end of open wedge [30]. However, since all failure points were located beneath the lateral hinge, the reason of failure was the insufficient mechanical strength of residual bone. If early weight bearing is suggested for better bone healing after medial owHTO surgery, the reinforcement by supplemental screw insertion can improve the structural mechanical durability, and share the physiological load and provide immediate weight bearing capability to realize the goal of ERAS.

The posterior slope of the tibial plateau is influential to knee joint function and joint contact force. High varus correction and lateral hinge fracture are risk factors for loss of reduction (increased posterior slope) after owHTO surgery, validated by Jacobi et al. in a cadaveric model [31]. This issue has not been discussed in the aforementioned practical biomechanical studies. In the finite element study by Yang et al., the conventional medial owHTO technique represented an obvious trend that greater compressive displacement was found at the posteromedial tibial plateau, either in the simulated load for upright standing or sit-to-stand phase. When a supplemental screw was inserted, the posteromedial tibial plateau was supported and showed a recovery of stability by 51.9–57.5% comparing to the loss of stability by plate only (100%) [21]. In the current study, at the 4^th^ load level, the group 1 further increased the posterior slope by 4.17 degrees in average compared to the intact group, which is apparently larger than the group 2 (0.76 degrees in average). Taking the full loss of posterior slope by 100% when only the bone plate was applied in the group 1, the group 2 preserved the posterior slope by 81.8% (i.e. only 18.2% loss of reduction). When considering varus correction loss, the supplemental screw insertion also restored the stability to 83.2%. The evidence provided in the current study helped to confirm that the strong support by a supplemental screw can maintain the structural stability without jeopardizing the safety of implanted components in medial owHTO surgery.

### Limitations

Some limitations of the current study were noted:

- Due to limited research resources, the sample size was not very large. In our current study, the intergroup differences were not highly significant. The specimens were carefully prepared to reduce the possible influence from human factors. However, some other important physiological loading conditions such as torsion and bending moment were not simulated in the current study. A further investigation may be necessary to comprehensively realize the biomechanical performance of the supplemental screw in medial owHTO model.
- Although the sawbone specimen has been validated for its mechanical behaviors compared to cadaveric bones, the property of sawbone is more like a plastic material lacking flexibility. When performing the opening of the osteotomy site for varus correction, it is very difficult and technically demanding to preserve an intact lateral hinge. Some of the studies created the medial owHTO by removing the wedge from a normal tibial sawbone instead of expanding the gap for varus reduction [25, 32]. In order to correctively reproduce the medial owHTO protocol, we chose the tibial sawbone with varus deformity at tibial plateau for the test. Although the lateral cortex remained intact, it was unknown if a microfracture or related damage has been created within the hinge structure.
- The failure observation was confirmed visually and acoustically. Therefore, the current study did not provide the exact number of failure cycles. Because the lowest frequency for visual confirmation was within 100 cycles, so the interval of 100 cycles were represented in the current study for describing the failure cycles.
- The test specimen was a stand-alone tibial sawbone without the lateral support by the fibula. The fibula shares around one-sixth of the load from the knee joint. The reduced lateral support may also influence the resistance to possible valgus moments.
- The pre-tension provided by the supplemental screw insertion was difficult to quantify. It is expected that a fully threaded screw may firmly stabilize the gap between the superior and the inferior bone fragments, while a partially threaded screw (ex. lag screw) may tend to close the gap and offers a secondary compression to the lateral hinge in medial owHTO surgery. Surgeons would decide the tightening level for the partially threaded screw referring to personal experience or preference. However, the artificial sawbone cannot provide similar feedback torque for surgeon. Further study for optimal strategy for supplemental screw insertion technique will be required.

## Conclusion

The current in-vitro biomechanical study encouraged the usage of supplemental screw in medial owHTO structure with improved mechanical strength, reduced loss of reduction, and better durability to dynamic cyclic load compared to the regular treatment without the supplemental screw. Further studies for more comprehensive biomechanical verifications, and optimizing surgical technique and for confirming clinical efficacies should be conducted in the future.

## Supporting information

S1 Fig Data for average structural stiffness. (XLSX)

S2 Fig Data for tibial plateau slope change in coronal plane. (XLSX)

S3 Fig Data for tibial plateau slope change in sagittal plane. (XLSX)
